# Conducting quantitative mask fit tests: application details and affecting factors

**DOI:** 10.3389/fpubh.2023.1218191

**Published:** 2023-07-13

**Authors:** Xiaodan Xu, Liangliang Zhao, Yong Zhu, Bing Du, Baoli Zhu, Hengdong Zhang, Lei Han, Xin Liu

**Affiliations:** ^1^Jiangsu Provincial Center for Disease Control and Prevention, Institute of Occupational Disease Prevention, Nanjing, China; ^2^Jiangsu Province Engineering Research Center of Health Emergency, Nanjing, China; ^3^Pudong New Area Center for Disease Control and Prevention, Shanghai, China; ^4^BASF-YPC Company Limited, Nanjing, China

**Keywords:** respiratory protective device, mask, quantitative fit test, fit factor, controlled negative pressure, condensation nuclei counting

## Abstract

**Introduction:**

Respirators chosen based on their assigned protection factor (APF) enable wearers to effectively reduce particulate matter concentrations to safe levels when used correctly. As a crucial factor in achieving the intended APF, the fit test has become a necessary procedure in respiratory disease protection.

**Methods:**

This study involved 225 participants who underwent a fit test using two reusable types of half masks and two types of full masks. Condensation nuclei counting (CNC) and controlled negative pressure (CNP) were performed.

**Results:**

The results revealed that the passing rate of full masks was higher compared to half masks. Specifically, the passing rate for the half masks and the full masks were 84.7 and 91.6%, respectively. Gender exerted a statistically significant effect on the passing rate. Nevertheless, age, educational background, and training exhibited relatively negligible effects. Certain movements, such as facing forward, were identified as key actions with strong correlation. Additionally, talking was considered a key action with a high failure rate due to instantaneous leakages. Most participants failed at the initial step of CNP, but nearly all of them passed the fit test using CNC.

**Discussion:**

Therefore, putting on full masks, especially for women, provides optimal protection during work. Furthermore, attention should be given to the displacement and deformation of the respirator during the key actions. When it comes to fit test methods, CNC was found to be more practical and comprehensive compared to CNP. Moreover, additional physiological characteristics, such as double chins, could be explored as potential influential factors.

## 1. Introduction

When considering various occupational hazards, inhalation poses a significant risk, with substances such as particulate matter, toxic gases, and vapors being major causes of human injury. These hazards can lead to conditions such as pneumoconiosis, occupational poisoning, and other occupational diseases (1–4). For instance, the COVID-19 outbreak was primarily caused by the virus being carried on particulate matter and transmitted to people through contact (5–9). As of the end of 2021, occupational pneumoconiosis cases continued to rank first among occupational diseases (10). Despite the presence of engineering protection facilities, the concentration of particulate matter in many workplaces cannot be reduced to safe levels on certain occasions. Hence, respirators are indispensable in preventing harm to workers by respirable gases or particulate matter. A respirator worn correctly can trap most particles, thus delivering purified air to the wearer (11–14).

There are various types of respiratory protective equipment (RPE). The assigned protection factor (APF) serves as the basis for determining the level of protection provided by masks, as stipulated in the recommended China national standard GB/T 18664-2002, “Selection, Use, and Maintenance of Respiratory Protective Equipment” (15). The APF refers to “a respiratory protective device or class of respirators that, when used correctly, is expected to reduce the concentration of air pollutants to an acceptable level” (16–18). The Occupational Safety and Health Administration of the United States (OSHA) standards explicitly state that “APFs are effective only if the employer implements an ongoing respiratory protection program that includes training, fit test, maintenance, and usage requirements” (19). Therefore, the respirator fit test and proper wearing of RPE are prerequisites for achieving the intended APF value.

The fit test for respirators can be performed using various qualitative and quantitative methods (20). Quantitative testing methods are often applied to various devices. The respirator fit test performed by PortaCount of TSI and MT of SIBATA yielded similar results by quantitative methods (21). The relative impact of fit test exercises and mask donning on respirator fit was measured using controlled negative pressure and an ambient aerosol fit test system. Donning was found to have a greater effect on respirator fit compared to fit test exercises (22). Recent studies have focused on respirator fit tests to evaluate the effects of wearing a breathing apparatus, indicating a new trend (23–25). Further research is needed on the fit test procedures and application details of various respirators. Thus, this article aimed to intensively study the influencing factors of the respirator effectiveness and key actions in the fit test through the test data of two technological paths.

## 2. Methods

### 2.1. Subjects and operators

A total of 225 chemical plant operators and maintenance and laboratory personnel were selected as subjects. Each subject could perform multiple tests. The gender, age, educational background, and training of the subjects were collected. Persons being fit tested were to be medically cleared to wear the respirator prior to fit testing, while subjects with respiratory diseases were to be excluded. Persons to be fit tested were to understand the test procedure and could complete the test process independently.

The fit test operators were to be familiar with respirator fit testing, inspection, cleaning, maintenance, and storage in the respiratory protection program.

### 2.2. Respirators and testers

In this study, four types of RPE currently produced by MSA Safety Incorporated were selected: two half masks (a 410 air-purifying respirator and 420 air-purifying respirator) and two full masks [a 3S air-purifying respirator and Ultra Elite self-contained breathing apparatus (SCBA)]. The 3S air-purifying respirator includes the 3S (black rubber frame) and 3S economy (white frame) types. An air-purifying respirator is designed with a filter, cartridge, or canister, which efficiently eliminates specific air contaminants by filtering air through a purification element. A self-contained breathing apparatus is an atmosphere-supplying respirator for which the breathing air source is designed to be carried by the user.

The QuantiFit tester by OHD (Hoover, Alabama, United States) and PortaCount Pro8038 fitting tester by TSI (Shoreview, Minnesota, United States) were used to perform the test. The testers had undergone annual factory calibration authorized service facility.

**Figure 1:**
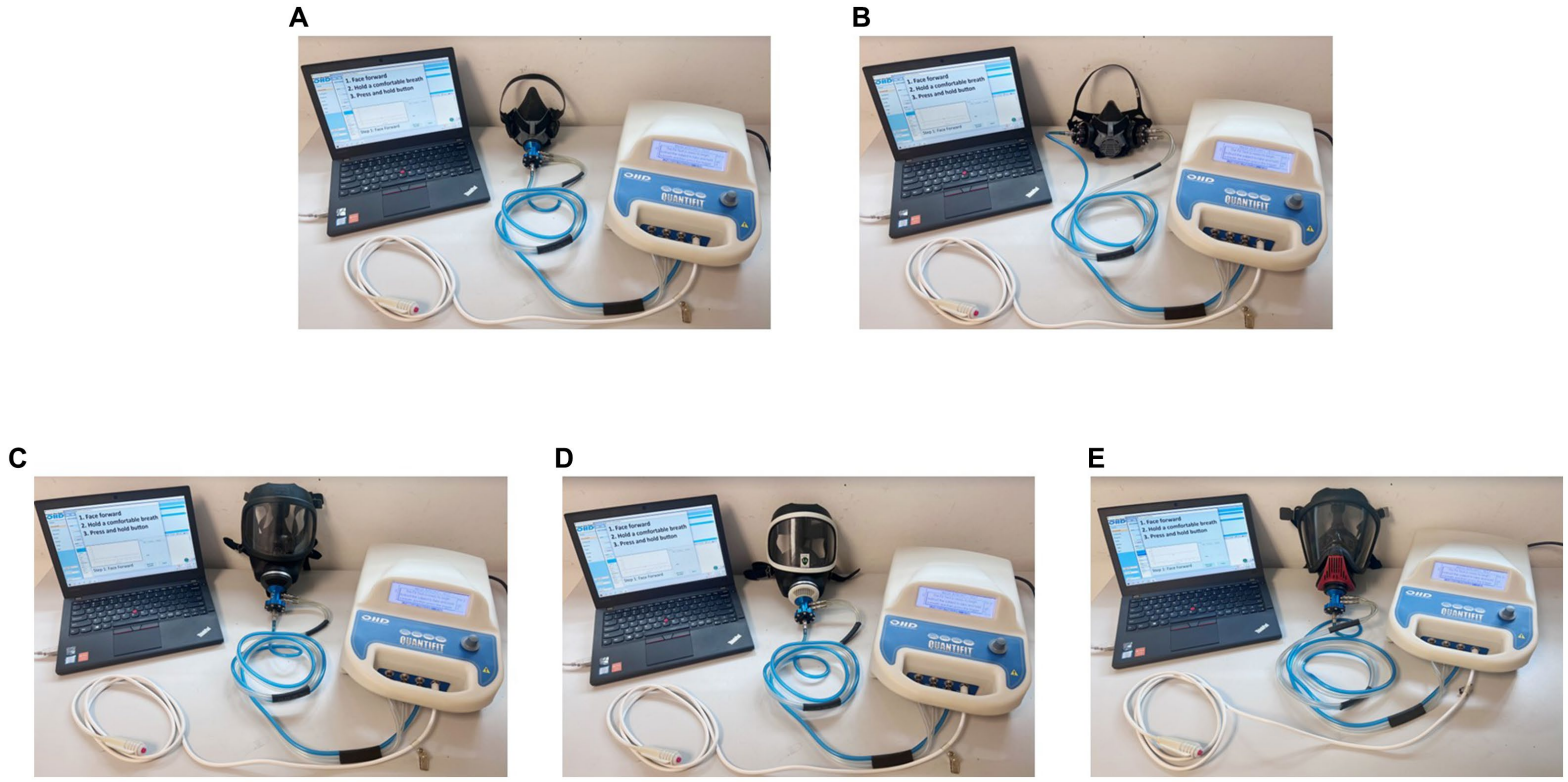
Respirators connected with the QuantiFit. **(A)** 410 air-purifying respirator; **(B)** 420 air-purifying respirator; **(C)** 3S air-purifying respirator—black rubber frame; **(D)** 3S air-purifying respirator—white frame; **(E)** Ultra Elite self-contained breathing apparatus.

The QuantiFit functions by creating and maintaining a negative pressure in the respirator. Once the adapter valve is closed, sealing the respirator, the QuantiFit removes air from the respirator until the challenge pressure is reached. During the fit test, the QuantiFit measures exactly how much air the instrument removed from the respirator after reaching the challenge pressure. Air inhalation or exhalation, even slightly, creates dramatic changes within the respirator. Actions such as swallowing or opening the mouth can adversely affect the pressure sensor and cause tests to fail. Daily verification is required for each day of testing. This verification measures the leak rate of the leak orifice on the tube assembly at various pressure levels and confirms that the diaphragm pump and other processes are working correctly. The QuantiFit with respirators is displayed in Figure 1.

**Figure 2:**
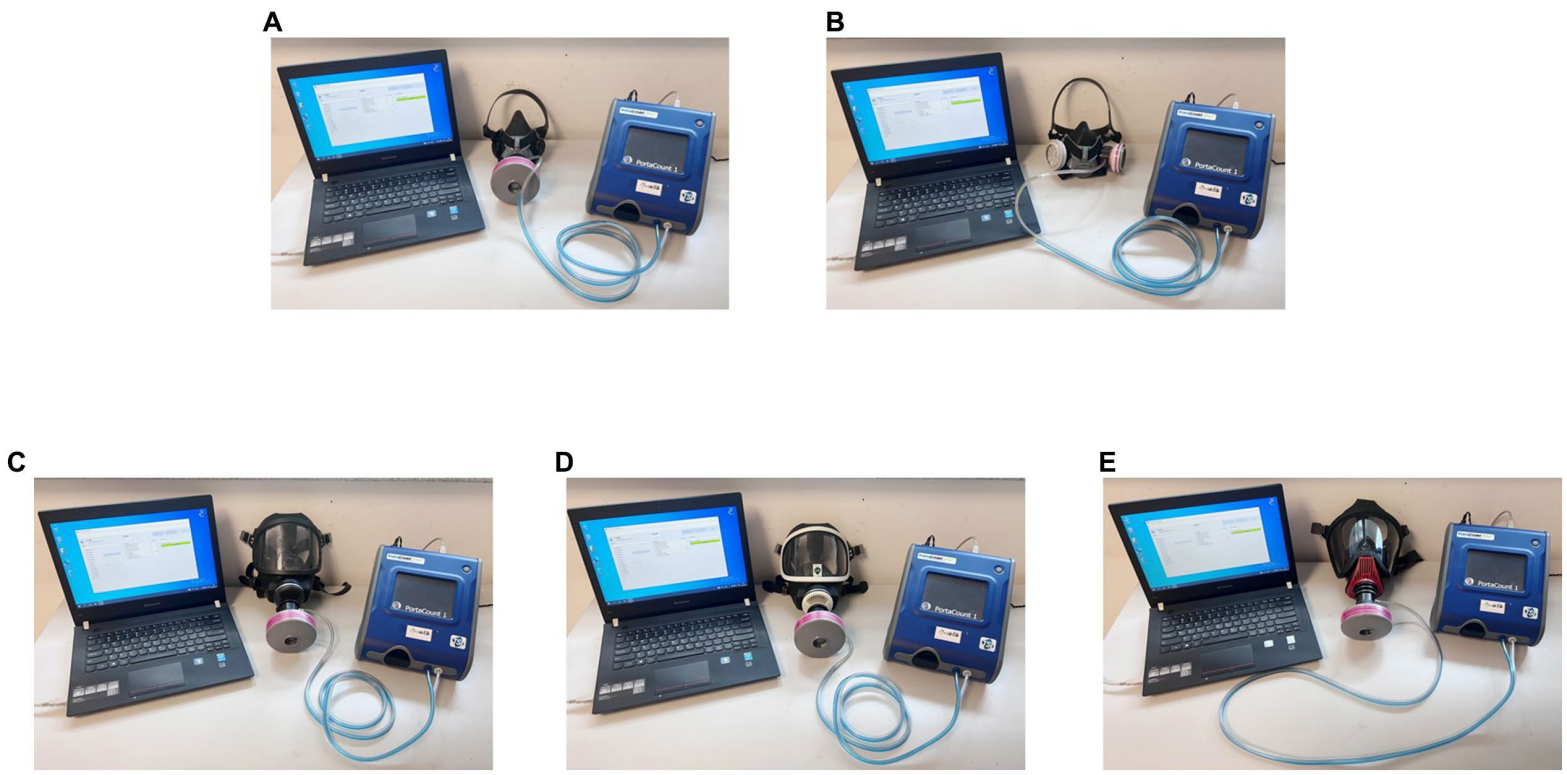
Respirators connected with the PortaCount Pro8038. **(A)** 410 air-purifying respirator; **(B)** 420 air-purifying respirator; **(C)** 3S air-purifying respirator—black rubber frame; **(D)** 3S air-purifying respirator—white frame; **(E)** Ultra Elite self-contained breathing apparatus.

The PortaCount Pro8038 fitting tester is designed to operate using the microscopic particles in the ambient air. It can measure particle concentrations and fit factors when generated aerosols (such as corn oil, salt, or ambient air) are used; however, these aerosols may cause the PortaCount Pro8038 to need more frequent cleaning and calibration checks. Instruments with adapters can perform daily verification, which determines if the tester is working correctly and if the concentration of particles in the ambient air is sufficient to conduct fit testing. The effective operation of the instrument is limited by aerosol concentrations. Completed daily checks provide confidence that the test results are reliable. Moreover, it is very important to instruct individuals not to smoke for at least 30 min prior to fit testing. The PortaCount Pro8038 with respirators is displayed in Figure 2.

### 2.3. Test methods

OSHA-accepted quantitative fit tests were used in this study: controlled negative pressure (CNP) and condensation nuclei counting (CNC).

CNP enables the direct measurement of air flow into the mask under negative pressure conditions (20), which is applicable only to re-usable respirators, such as the four types of masks used in this study. The fit test procedure took about 2–3 min and consisted of several parts. First, the mask was connected to the QuantiFit tester and was put on correctly. Second, the test subjects performed a specific movement and then held their breath for 10 s. The fit factor (FF) was given by the tester after breath holding (Equation 1). Third, five movements were prescribed: facing forward, bending over (stayed), shaking the head, wearing of the mask again, and wearing of the mask again. Fourth, the fit test was ended, and the mask was taken off. The instrument finally presented the overall FF (Equation 2).

CNC measures the aerosol concentration inside and outside the breathing zone of the respirator using a probe (21, 24, 26, 27). The aerosols measured inside the respirator are assumed to be the result of face seal leakage. Ambient air aerosol was used in this study. The CNC test period is longer than that of CNP by about 8–9 min. First, the mask was connected to the PortaCount Pro8038 fitting tester and put on correctly. Second, the test subjects performed a specified movement repeatedly for 60 s. The instrument displayed the FF at the end of 60 s (Equation 3). Third, eight movements were prescribed: normal breathing, deep breathing, moving the head side to side, moving the head up and down, talking, grimacing, bending over (repetitive), and normal breathing. In particular, the grimace lasted for 15 s with no FF. Fourth, the fit test was ended, and the mask was taken off. Here, to assess whether the mask would re-close to the face after leakage, we deliberately carried out grimacing to create leaks. However, this action would not be considered in the calculation of the overall FF. After the remaining seven actions had been completed, the instrument showed the overall FF (Equation 2).


(1) $$\mathrm{FF}=\frac{\mathrm{Modeled breathing rate}\;\left(\frac{\mathrm{cc}}{\min}\right)}{\mathrm{Measured leak rate}\;\left(\frac{\mathrm{cc}}{\min}\right)}$$

(2) OverallFF=N1FF1+1FF2+⋯+1FFN

(3) $$\mathrm{FF}=\frac{\mathrm{Aerosol count concentration outside the mask}\;\left(\frac{\mu g}{m^{3}}\right)}{\mathrm{Aerosol count concentration inside the mask}\;\left(\frac{\mu g}{m^{3}}\right)}$$

where *N* is the number of movements and FFn is the fit factor for the nth movement.

OSHA requires a fit factor of at least 100 for half masks and 500 for full masks when using an OSHA-accepted quantitative fit test method. If the overall FF of a half mask is ≥100 (required FF, RFF) and the overall FF of a full mask is ≥500 (RFF), the respirator fit test is considered successful. Consequently, the mask is deemed suitable for the wearer; otherwise, it is deemed unsuitable.

### 2.4. Statistics

The original data from the test software were exported and organized using Excel. Statistical analysis was performed using SPSS 27.0.1. Chi-square test was used to analyze the influencing factors of the fit test, while multiple linear regression was employed to analyze the FFs.

## 3. Results and discussion

### 3.1. Respirator fit test passing rate

The test categories are displayed in Table 1. The CNP and CNC test results for the different respirators were analyzed. The passing rates for the half masks and full masks were 84.7 and 91.6%, respectively. The overall passing rate followed the order: half masks < full masks. The difference was statistically significant (*p* < 0.05).

**Table 1:** Passing rate of four respirators.

Respirator	Testing number^*^ (CNP)	Testing number^*^ (CNC)	Total testing number^*^	Passing numbers^*^	Passing rate	Passing rate (half masks)	Passing rate (full masks)
410 air-purifying respirator	56	31	87	71	81.6%	84.7%	–
420 air-purifying respirator	109	39	148	128	86.5%		
3S air-purifying respirator	200	39	239	215	90.0%	–	91.6%
Ultra Elite SCBA	97	8	105	100	95.2%		

*Cumulative numbers > total number. One run involved multiple tests.

Full masks provide better coverage by eliminating exposed areas such as the cheekbones and bridge of the nose. Therefore, full masks can meet the fitting requirements and achieve the desired APF more easily. A worker wearing a half mask will be extremely vulnerable to exposure to toxic environments when the mask is put on without a fit test.

Those who failed in the first test were asked to participate in the second test after receiving intervention. The main interventions included re-explaining the test method, a mask-putting-on demonstration, and changing the mask size if necessary. In the secondary test, eight subjects passed after the intervention. Among them, three passed the 420 air-purifying respirator test, two passed the 3S air-purifying respirator test, and three passed the Ultra Elite SCBA test. They increased the passing rate for the half masks and the full masks to 86.0 and 93.0%, respectively. The overall passing rate slightly increased after the intervention.

### 3.2. Affecting factors

In this article, the factors of gender (man and woman), age (≤45 and >45 years old), education background (below high school; high school or above), and training (yes or no) were investigated for their potential impact on the respirator fit test (Table 2). We only considered the assigned gender at birth in this part. A preliminary chi-square test was conducted to determine whether it had an effect on the test results (Table 3) (28). A chi-square test showed that gender had a *p* value < 0.05.

**Table 2:** Numbers of various factors.

Factors	Categories	Passing number^*^	Failing number^*^
Gender	Man	406	55
	Woman	31	10
Age	≤45	212	34
	>45	225	31
Education background	Below high school	42	9
	High school or above	395	56
Training	Yes	404	60
	No	33	5

*Cumulative numbers > total number. One run involved multiple tests.

**Table 3:** Effects of factors on respirator fit test.

Factors	*χ*^2^	*p*
Gender	5.186	0.023
Age	0.326	0.568
Education background	1.112	0.292
Training	0.002	0.968

The results revealed a significant influence of gender on the passing rate of the respirator fit test. In this study, we found that the passing rate of the men participants was 88.1%, while the passing rate of the women was 75.6%. As presented in Table 4, women generally exhibited a lower passing rate in the respirator fit test compared to men. Some participants tested different types of masks, so the total number of tests recorded exceeded the total number of participants. Moreover, women tended to choose smaller respirators in the testing process. After trying out different sizes of masks, a majority of women preferred the S-size. In terms of the low passing rate for women, a plausible reason may be the combined effect of having a thin face and sharp chin, which finally led to face seal leakage in the respirator fit test. Furthermore, in the CNP test, the subjects must hold their breath to maintain the mask’s negative pressure. The women tended to hold their breath for a shorter period of time, which was also a reason for the low passing rate.

**Table 4:** Effects of gender on respirator fit test.

Respirator	Man			Woman		
	Test number^*^	Pass number^*^	Passing rate	Test number^*^	Pass number^*^	Passing rate
Half masks	155	125	80.6%	23	17	73.9%
Full masks	306	281	91.8%	18	14	77.8%
Total	461	406	88.1%	41	31	75.6%

*Cumulative numbers > total number. One run involved multiple tests.

Meanwhile, age, education background, and training demonstrated negligible influence on the passing rate. Consequently, efforts to improve the protective effectiveness of respirators should primarily focus on physiological characteristics, such as gender, facial characteristics, and so on.

### 3.3. Key actions

#### 3.3.1. Key actions with strong correlation

From the introduction of the test methods, it is evident that the method consists of several test actions. Each test action is a step in the fit test, and it can be considered that a CNP test contains 5 steps, while a CNC test contains 8 steps.

There are two kinds of key actions. One key action is strongly correlated with the final test result, and it can be found by the correlation between the steps’ FFs and overall FF. In this article, we define it as “key actions with strong correlation.” An effective and accepted statistical method for analyzing correlations is multiple linear regression (29). In consequence, multiple linear regressions for the CNP and CNC tests were presented to explore the key actions with strong correlation. We treated the steps’ FFs as the independent variables and the overall FF as the dependent variables in multiple linear regression, with consideration of the 3S air-purifying respirator as an example. In multiple linear regression, the smaller the *p* value, the stronger the correlation between the variables. In this article, a *p* value < 0.001 was considered as the criterion for determining the key actions.

Prior to the analysis, necessary regression checks were performed on the CNP data. The rules were as follows: Step 1: facing forward; Step 2: bending over (stayed); Step 3: shaking the head; Step 4: wearing of the mask again; and Step 5: wearing of the mask again.

The collinearity diagnostic criteria show that the greater the tolerance of the independent variables, the weaker the multicollinearity between the variables. A tolerance greater than 0.1 is acceptable, indicating that there is no multicollinearity problem between the independent variables (30, 31). As shown in Table 5, the FF tolerance values in collinearity statistics were all greater than 0.1, indicating no significant multicollinearity issues with FFs. The Durbin–Watson test yielded a value of 1.888, indicating that the data residuals can be considered independent.

**Table 5:** Check of multiple linear regression (CNP).

Model^a^	Steps	Tolerance	Durbin–Watson
(Constant)			1.888
Step 1 FF	Facing forward	0.919	
Step 2 FF	Bending over (stayed)	0.833	
Step 3 FF	Shaking the head	0.867	
Step 4 FF	Wearing of the mask again	0.791	
Step 5 FF	Wearing of the mask again	0.840	

aDependent variable: overall FF.

**Figure 3:**
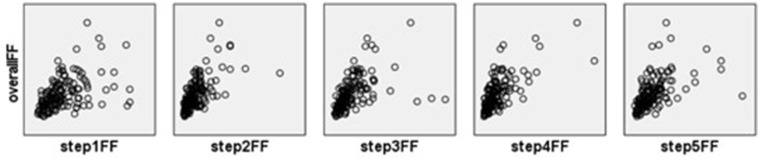
Scatter plots of steps’ FFs and overall FF (CNP).

**Figure 4:**
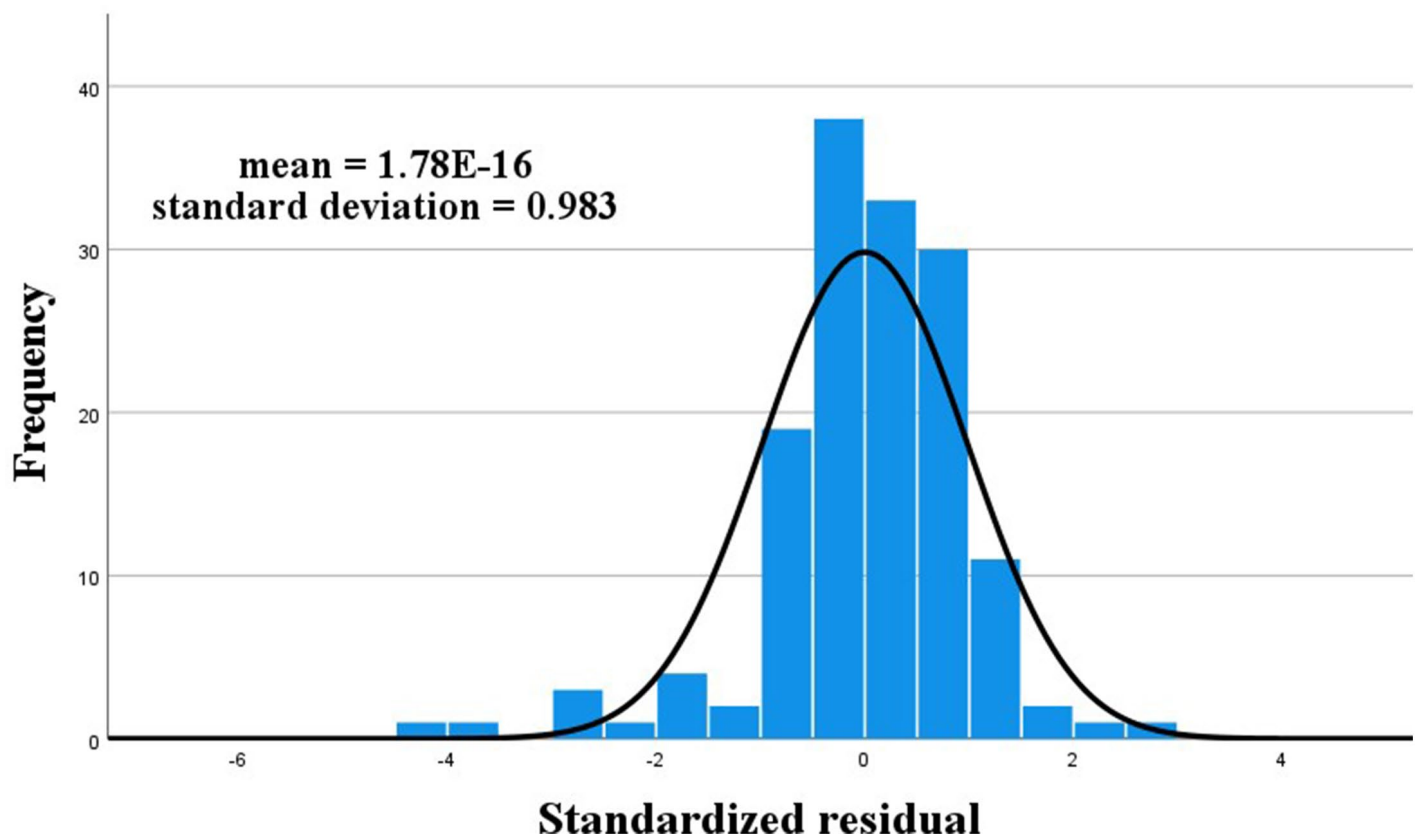
Histogram of residuals in CNP.

**Figure 5:**
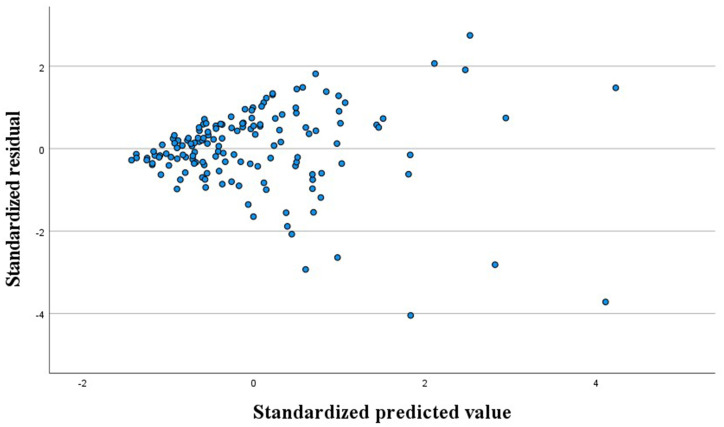
Scatter plot of residuals in CNP.

The overall FF increased and decreased according to the steps’ FFs in Figure 3, suggesting a simple linear relationship between the steps’ FFs and overall FF. The histogram of residuals, as shown in Figure 4, demonstrated a normal distribution with an average value of 0 and a standard deviation of 1, indicating a good fit to the normal distribution. As shown in Figure 5, the regression-standardized residuals were distributed around 0, with symmetrical data points above and below. Consequently, the data residuals met the assumptions of the homogeneity of variance. Based on the above checks, the steps’ FFs and overall FF can be analyzed via multiple linear regression.

Similar checks confirmed that CNC also satisfied the conditions for multiple linear regression, enabling the identification of key actions in CNC. The following rules were applied: Step 1: normal breathing; Step 2: deep breathing; Step 3: moving the head side to side; Step 4: moving the head up and down; Step 5: talking; Step 6: bending over (repetitive); and Step 7: normal breathing. Since the grimace step does not have an *F* value, this step was not included in multiple linear regression, and seven steps were listed in the CNC test.

Multiple linear regression analyses were carried out for the CNP and CNC data. The results of the multiple linear regression for CNP revealed a significant regression equation, with an *F* value of 123.349 and *p* < 0.05. The steps’ FFs can account for 80.7% of the variation in the overall FF (Table 6). The *p* values of the steps indicated that all of them had a positive and strong effect on the overall FF. Thus, facing forward, bending over (stayed), shaking the head, and wearing of the mask again were all key actions with strong correlation.

**Table 6:** Coefficient of the regression model (CNP).

Model^a^	Steps	Beta	*t*	*P*	*F*	*R*^2^ (adjusted)
(Constant)			3.995	0.000	123.349	0.807
Step 1 FF	Facing forward	0.258	6.814	0.000		
Step 2 FF	Bending over (stayed)	0.347	8.715	0.000		
Step 3 FF	Shaking the head	0.182	4.658	0.000		
Step 4 FF	Wearing of the mask again	0.381	9.337	0.000		
Step 5 FF	Wearing of the mask again	0.242	6.118	0.000		

a Dependent variable: overall FF.

The results of the multiple linear regression for CNC indicated a significant regression equation, with an *F* value of 9.724 and *p* < 0.05. The steps’ FFs can explain 61.6% of the variation in the overall FF (Table 7). Step 4 FF (*p* < 0.001) had a positive and strong impact on the overall FF. During the 60 s up-and-down head movement, the neck surface experienced frequent squeezing and stretching, increasing the likelihood of gas leakage at the lower end of the mask. Based on this finding, only moving the head up and down was considered a key action with strong correlation in CNC.

**Table 7:** Coefficient of the regression model (CNC).

Model^a^	Steps	Beta	*t*	*p*	*F*	*R*^2^ (adjusted)
(Constant)			2.568	0.015	9.724	0.616
Step 1 FF	Normal breathing	0.039	0.290	0.774		
Step 2 FF	Deep breathing	0.475	2.237	0.033		
Step 3 FF	Moving the head side to side	−0.178	−1.198	0.240		
Step 4 FF	Moving the head up and down	0.713	4.418	0.000		
Step 5 FF	Talking	0.144	0.601	0.552		
Step 6 FF	Bending over (repetitive)	−0.553	−1.958	0.059		
Step 7 FF	Normal breathing	0.249	0.815	0.421		

a Dependent variable: overall FF.

Through the multiple linear regression analysis of the CNP and CNC test data, we can conclude that the key actions with strong correlation are facing forward, bending over (stayed), shaking the head, wearing of the mask again, and moving the head up and down. When applying a respirator, these key actions need to be given high attention to ensure that the wearer’s breathing environment is sustainable and safe.

#### 3.3.2. Key actions with high failure rate

Another key action has a high failure rate, and we define it as “key actions with high failure rate” in this article. There will be several instantaneous leakages during key action with a high failure rate, which is usually the main reason for the high failure rate of the step. Instantaneous leakage refers to temporary air leakage that occurs despite resealing, leading to a poor sealing situation. In cases where a fit test ultimately passed despite failing one or two steps, key actions with high failure rate were observed. As shown in Table 8, about one-third of the failure steps can be attributed to “Talking.” Talking produces multiple instantaneous leakages, which can result in step failure, and is not conducive to the effect of respiratory protection. This finding aligns with the conclusion drawn by Sietsema M et al., who reported that talking can disrupt the fit of a respirator due to facial movements that may dislodge the facepiece (26). The high step failure rate attributed to instantaneous leakage highlights the significance of considering talking as a key action in the fit test.

**Table 8:** Subjects’ failure in steps.

Failed steps	No. of subjects	Proportion
Facing forward (CNP)	0	0%
Bending over (stayed, CNP)	0	0%
Shaking the head (CNP)	0	0%
Wearing the mask again (CNP)	0	0%
Wearing the mask again (step 5, CNP)	1	3.0%
Normal breathing (CNC)	2	6.5%
Deep breathing (CNC)	2	6.5%
Moving the head side to side (CNC)	9	29.0%
Moving the head up and down (CNC)	2	6.5%
Talking (CNC)	11	35.5%
Bending over (repetitive, CNC)	2	6.5%
Normal breathing (CNC)	2	6.5%
Total	31	100%

### 3.4. Applicability

Among the participants who failed the test in CNP, more than half of them discontinued the test at the first step, indicating their unsuitability for this particular method (Table 9). This outcome highlights the limitations of the CNP test despite its speed and accuracy. The test requirements, such as breath holding, pose challenges that some individuals are unable to meet. Interestingly, in the experiments conducted, the participants who dropped out of the CNP test were often able to pass the CNC test easily. However, those who failed the CNC test often failed the CNP test as well. In addition, the CNC test includes any tight-fitting respirator and a wide variety of actions, which is more realistic. This suggests that people generally exhibit higher adaptability to the CNC method in actual situations.

**Table 9:** Subjects’ terminating steps (CNP).

Terminating steps	No. of subjects	Proportion
Step 1: Facing forward	48	73.8%
Step 2: Bending over (stayed)	8	12.3%
Step 3: Shaking the head	8	12.3%
Step 4: Wearing the mask again	1	1.6%
Step 5: Wearing the mask again	0	0%
Total	65	100%

## 4. Conclusion

This study focused on the respirator fit test with four kinds of masks using QuantiFit and PortaCount Pro8038. The experiments involved 225 participants and presented the CNP and CNC test systems. The results indicated that putting on full masks had a higher passing rate compared to half masks. The passing rates for the 410 air-purifying respirator, 420 air-purifying respirator, 3S air-purifying respirator, and Ultra Elite SCBA were found to be 81.6, 86.5, 90.0, and 95.2%, respectively. Specifically, the passing rates for the half masks and the full masks were 84.7 and 91.6%, respectively. Interventions were shown to slightly improve the passing rate. Through chi-square tests, it was determined that gender significantly influenced the passing rate. The low passing rate among women was attributed to their facial features and shorter breath-holding time. Key actions were identified using correlation and step failure rate analysis. Based on the correlation of the steps’ FFs and overall FF, the key actions with strong correlation were identified as facing forward, bending over (stayed), shaking the head, wearing the mask again, and moving the head up and down. Additionally, talking was considered a key action with a high failure rate due to the occurrence of several instantaneous leakages. In terms of fit test methods, CNC was found to be more practical and comprehensive compared to CNP and should be the first choice for occupational safety and health practitioners.

Moreover, the presence of facial hair, such as beards and certain hairstyles, can affect the effectiveness of face masks (32, 33). Any external objects between the face and the respirator will interfere with the tightness, and it is recommended to remove foreign objects and keep the face clean and smooth before putting on a mask or performing a fit test. When exploring meaningful influencing factors, it is important to consider facial features such as the double chin. Furthermore, attention should be given to the displacement and deformation of the respirator during the key actions. Improper mask wearing can result in wearers being exposed to excessive or toxic particulate matter. Therefore, regular fit tests and the use of various masks, especially smaller sizes for women, are recommended to ensure optimal protection.

## Data availability statement

The raw data supporting the conclusions of this article will be made available by the authors, without undue reservation.

## Ethics statement

The studies involving human participants were reviewed and approved by Ethical Committee of Jiangsu Provincial Center for Disease Control and Prevention (JSJK2022-B002-01). The patients/participants provided their written informed consent to participate in this study.

## Author contributions

All authors listed have made a substantial, direct, and intellectual contribution to the work and approved it for publication.

## Funding

This work was funded by the Jiangsu Provincial Social Development Program of Key R&D Project (BE2022803) and Jiangsu Provincial Key Medical Discipline (ZDXK202249).

## Conflict of interest

BD was employed by BASF-YPC Company Limited.

The remaining authors declare that the research was conducted in the absence of any commercial or financial relationships that could be construed as a potential conflict of interest.

## Publisher’s note

All claims expressed in this article are solely those of the authors and do not necessarily represent those of their affiliated organizations, or those of the publisher, the editors and the reviewers. Any product that may be evaluated in this article, or claim that may be made by its manufacturer, is not guaranteed or endorsed by the publisher.
